# Diagnosis and Treatment of Atraumatic Splenic Rupture: Experience of 8 Cases

**DOI:** 10.1155/2019/5827694

**Published:** 2019-01-28

**Authors:** Jian Liu, Yanyu Feng, Ang Li, Chunqing Liu, Fei Li

**Affiliations:** ^1^Department of General Surgery, Xuanwu Hospital, Capital Medical University, Number 45, Changchun Street, Beijing 100053, China; ^2^Department of General Surgery, Daxing Teaching Hospital, Capital Medical University, Number 26, Huangcun West Street, Beijing 102600, China

## Abstract

Atraumatic splenic rupture (ASR) is rare but life threatening. In this study, we retrospectively described our experience on the diagnosis and treatment of 8 patients (male: 6; female: 2; mean age: 49.6) with ASR. ASR accounted for 3.2% (8/251) of the splenic ruptures. The clinical presentation of ASR was similar to traumatic splenic rupture (TSR). The sensitivity of ultrasound and contrast-enhanced computed tomography (CECT) in ASR diagnosis was 57.1% and 85.7%, respectively. According to the classification of the American Association for the Surgery of Trauma (AAST), 2 cases were classified as grade II splenic ruptures, 4 cases were classified as grade III ruptures, 1 case was classified as grade IV rupture, and 1 case was not classified. All the spleens became swollen, and hematomas were observed in 6 patients. Total splenectomy was recommended in most cases. At least 62.5% (5/8) of the patients with 7 etiological factors belonged to “atraumatic-pathological splenic rupture.” Local inflammation and cancer were the most common etiological factors.

## 1. Introduction

Spleen rupture is mostly caused by trauma. However, in some rare cases, it also occurs without obvious trauma, which is called atraumatic splenic rupture (ASR) or spontaneous splenic rupture. ASR is often life threatening due to the delay of diagnosis and treatment.

ASR is uncommon, and little is known about its characteristics. There is still a lack of related guidelines or a standard of diagnosis and treatment for ASR. In this study, we described the characteristics of ASR patients from two university teaching hospitals with the goal of increasing the knowledge of ASR, especially its underlying etiological factors, diagnosis, and management methods.

## 2. Methods

We retrospectively identified patients with splenic rupture from two university teaching hospitals between January 2004 and December 2014. The classification criteria of a splenic rupture was based on the American Association for the Surgery of Trauma (AAST) [1]. The criterion of “splenomegaly” was spleen exceeding 200 g in weight or 110 × 70 × 50 mm (in vivo) in size [2].

The exclusion criteria are as follows: (1) patients with a left upper quadrant trauma history and (2) patients with a splenic injury during abdominal surgery. Splenic ruptures after colonoscopy were not excluded.

Baseline data included sex, age, and clinical presentation. Data from laboratory tests, imaging examinations, surgical information, and pathological examinations were also collected. We attempted to reveal the possible etiological factors leading to ASR.

## 3. Results

A total of 251 cases of splenic rupture were diagnosed during 11 years, of which 8 (3.2%) ASR patients were identified. No patient died during the hospital stay. The major characteristics of the 8 patients are shown in Table 1.

All the patients suffered from more or less abdominal pain, and Kehr's sign (acute pain at the tip of the shoulder due to the presence of blood or other irritants in the peritoneal cavity) was observed in two cases. Concomitant symptoms included nausea, abdominal distension, disturbance of consciousness, and intestinal obstruction. The symptoms of half of these patients were accompanied with peritonitis and shock.

**Figure 1:**
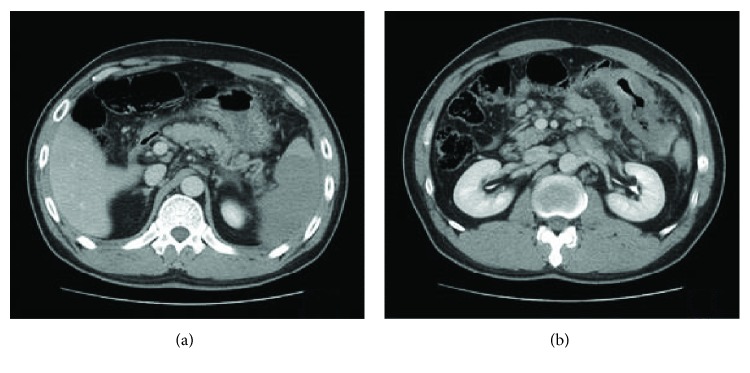
CT scan of patient no. 5. (a) Axial section of the CT scan showed an infarction of the lower pole of the spleen on admission. (b) Axial section of the CT scan showed a colon tumor of the splenic flexure.

The mean lowest hematocrit was 29.8% in seven patients who received surgery. The alanine aminotransferase (ALT), aspartate aminotransferase (AST), and creatinine (Cre) levels were significantly elevated in patient no. 2, which decreased to normal levels before discharge. Amylase (AMY) was mildly elevated in two patients whose conditions were complicated with autoimmune pancreatitis (AIP) and pancreatic cancer, respectively. Carbohydrate antigen 199 (Ca 199) was elevated in one patient with pancreatic cancer. The time from admission to surgery ranged from 2 hours to 5 days, and 71.4% (5/7) of the surgeries were performed within 8 hours. Patient no. 5 received conservative treatment on admission (Figure 1(a)) but became hemodynamically unstable after two days and received emergency surgery finally. Patient no. 7 received two surgeries, which occurred on the fifth day after the first admission and six months later.

**Figure 2:**
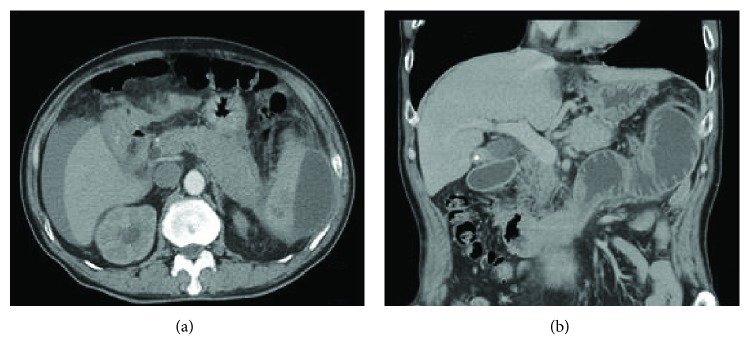
CT scan of patient no. 4. (a) Axial section of the CT scan showed a hematoma in the lower pole and lateral spleen area combined with the enlargement of the body and tail of the pancreas and free fluid around the liver and spleen. (b) Coronal section of the CT scan showed an obstruction of the small intestine.

Besides spleen rupture, we also found 1 case of hepatic aneurysm rupture, 1 case of perisplenic extensive adhesion, 1 case of colon tumor of splenic flexure (Figure 1(b)), 1 case of hepatic cirrhosis, 3 cases with abnormalities in the body and tail of the pancreas (Figure 2(a)), and 1 case of tortuosity and expansion of the gastroepiploic vein during operation. Three patients recovered eventually, and the remaining cases developed complications, including retroperitoneal hematoma, peritoneal abscess, deep vein thrombosis, pulmonary embolism, intestinal obstruction (Figure 2(b)), and secondary surgery.

Seven patients received pathological examination, and no pathological abnormality was identified in 42.8% (3/7) of the patients (patients no. 1, no. 2, and no. 3). The remaining 4 pathological changes included thrombosis in a splenic artery plus rupture and bleeding of a subcapsular hematoma (patient no. 4), massive infiltration of signet ring cell carcinoma in the spleen plus colon cancer (patient no. 5), spleen conforming to the changes of congestive splenomegaly plus chronic pancreatitis (patient no. 6), and mucinous adenocarcinoma in the pancreas (patient no. 7). Etiological factors of each patient were also listed in Table 1.

## 4. Discussion

The incidence rate of ASR has not been clarified. In this study, we showed that the incidence of ASR was 3.2% (8/251). The proportion of male ASR was 75% (6/8), and the mean age was 49.6 years, which is similar to previous reports [2–4].

The mortality of ASR is 12.2%-20% [2, 4, 5]. Splenomegaly, age above 40 years, neoplastic disorders, and delayed diagnosis or management are significantly associated with a fatal outcome [2, 5]. The mortality of ASR was 0 in this study, which was potentially attributed to our timely diagnosis and appropriate management.

There are several nomenclatures describing the rupture of the spleen without trauma, such as “atraumatic,” “pathological,” “spontaneous,” “idiopathic,” “true spontaneous,” and “occult” rupture [2]. Spontaneous splenic rupture was first described by Rokitansky (1861) and Atkinson (1874) [3], and it is also frequently used. However, it cannot distinguish a normal spleen from a spleen with pathological changes. “Atraumatic” is more accurate, and it can be classified as “atraumatic-pathological splenic rupture” and “atraumatic-idiopathic splenic rupture” according to the etiological factor and pathological changes in the spleen [2]. In this series, at least 5 patients (62.5%) with 7 etiological factors belonged to atraumatic-pathological splenic rupture. Because intra-abdominal aneurysm has the same pathogenesis, we suspect that patient no. 2 may suffer from a small splenic artery aneurysm, and the evidence of the artery aneurysm may be destroyed after the rupture [6]. If this was the case, there would be 6 (75%) “atraumatic-pathological splenic rupture” patients.

It sometimes takes a long time to identify the underlying etiological factors for ASR. In this series, etiological factors were identified in only 37.5% of ASR patients (3/8: anticoagulant, polycythemia vera, and splenic flexure colon tumor) before surgery and in 50% of ASR patients (4/8: chronic pancreatitis, hepatic cirrhosis, pancreatic cancer and AIP) during or after surgery, which was 41.7% and 27.8%, respectively, in Renzulli et al.'s research [2]. Other studies reported that up to 71% of the underlying causes of ASR with amyloidosis were unknown at the time of surgery [7]. In addition, some underlying etiological factors such as some viral infectious disorders may be missed ultimately because of the limitation of diagnosis technology and pathological examination. In our study, the proportion of local etiological factors was 71.4% (5/7), including inflammatory disorders, neoplastic disorders, and congestive splenomegaly derived from diseases of the perisplenic organs, which was higher than Renzulli's data (10.9%) [2]. There was no infectious etiological factor in the present study, while the proportion was 27.2% in Renzulli's data [2]. Such difference may be due to the small number of patients and differences in the geographic characteristics and population.

In this study, the sensitivity of ultrasound and contrast-enhanced computed tomography (CECT) in diagnosing ASR was 57.1% (4/7) and 85.7% (6/7), respectively, while the accuracy of CECT in diagnosing TSR is almost 100% [8]. To our knowledge, this is the first time that the sensitivity of ultrasound and CECT in diagnosing ASR was assessed, even though the evidence level of the data was low. CECT could also identify some etiological factors, such as the “enlargement of body and tail of pancreas” and “splenic flexure colon tumor” and some information implying surgery or nonsurgical treatment, such as “large free fluid in abdominal cavity” and “single subcapsular hematoma.” We propose that as long as the hemodynamic status is stable, CECT is necessary for patients with suspicious ASR.

All the spleens became swollen in this series, suggesting that the morphology of the spleens had been altered earlier by some pathological or non-pathological reasons. Therefore, if patients with abdominal pain are hemodynamically unstable and the spleen is swollen, ASR should be considered. Hematomas were found in all patients except one, indicating that the presentation may be secondary to the formation and rupture of a hematoma. Hematoma maybe caused by a pathological mechanism or tiny trauma, such as malignant cells [9].

In this series, we specified the rupture grade according to AAST which was not mentioned in most previous reports. Most ASR belonged to grade III or II. Treatment was based on hemodynamics, etiological factors, and CT grading. If the hemodynamic status is stable and the etiological factors are obvious, then nonsurgical treatment can be considered [10, 11]; however, the failure rate is relatively high [2]. In this study, one patient with grade II rupture, who had a history of polycythemia vera, successfully received nonsurgical treatment. If surgery is necessary, total splenectomy is suitable for most ASR. First, a pathological examination of the spleen and other abnormalities is helpful to identify underlying diseases. Second, most ASR patients belong to atraumatic-pathological splenic rupture, and the immunologic function of the spleen may have been lost and the removal of spleen will not increase the risk of an overwhelming postsplenectomy infection [2]. Third, the time from onset to surgery is usually long and the spleens have become edematous and fragile, which may increase the difficulty of splenorrhaphy. In this series, seven grade II-IV patients received surgery, and only one received organ-preserving surgery (subcapsular hematoma evacuation). Such proportion was 1.4% (9/660) in a previous review [2]. The spleen and perisplenic organs should be inspected carefully during operation to justify whether an additional operation or biopsy is necessary.

We performed laparotomy without the laparoscopic approach. Such choice was preferable because it was difficult to separate severe adhesion, excise the distal part of the pancreas, or excise a colon tumor under a laparoscope on emergency conditions. However, a laparoscope maybe a choice in some cases [12]. Other procedures were frequently associated to splenectomy, and the small number of patients who eventually recovered implied that ASR had higher surgical difficulty and worse outcome than TSR.

## 5. Limitation

The number of patients of this retrospective study is relatively small and thus cannot be representive for all ASR.

## 6. Conclusions

This study showed that ASR is rare. Local inflammation and cancer are the most common etiological factors. CECT, which has a high sensitivity, is often essential to diagnose ASR. Spleens become swollen and splenic hematomas can be observed in most cases. Intraoperative examination is helpful to find underlying abnormalities. Total splenectomy is recommended in most cases, and conservative treatment is applied to a few patients, depending on the etiological factors and hemodynamic status. ASR has higher surgical difficulty and worse outcome.

## Figures and Tables

**Table 1:** Characteristics of 8 patients with ASR.

Patient number	Sex	Age	Peritonitis	Shock	Hematocrit (%)	US	CECT	Splenomegaly	Hematoma	AAST classification	Surgical approaches	Etiological factors
1	M	34	Yes	Yes	31.7	Pos	Pos	Yes	Yes	III	Splenectomy	Unknown
2	M	52	Yes	Yes	25.6	Neg	NP	Yes	No	II	Splenectomy; repair of hepatic aneurysm	Unknown
3	F	63	No	No	39.2	Pos	Pos	Yes	Yes	III	Splenectomy	Unknown
4	M	63	No	Yes	16.1	Neg	Pos	Yes	Yes	III	Splenectomy	AIP, anticoagulant
5	M	41	Yes	Yes	20.2	Pos	Neg	Yes	Yes	IV	Splenectomy; left hemicolectomy	Splenic flexure colon tumor
6	M	37	Yes	No	42.1	NP	Pos	Yes	Yes	III	Splenectomy; distal pancreatectomy	Chronic pancreatitis, hepatic cirrhosis
7	M	38	No	No	33.1	Pos	Pos	Yes	Yes	NM	Subcapsular hematoma evacuation; pancreas biopsy (6 months later)	Pancreatic cancer
8	F	69	No	No	46.6	Neg	Pos	Yes	Yes	II	NP	Polycythemia vera

Abbreviations: US—ultrasound; CECT—contrast-enhanced computed tomography; AAST—American Association for the Surgery of Trauma; Pos—positive, Neg—negative, NP—not performed; NM—not mentioned; AIP—autoimmune pancreatitis.

## Data Availability

Research data was obtained from a retrospective analysis of individual case records, and only partial data can be provided in order to protect the patients' privacy.
